# Simultaneous Quantification of Diazepam and Dexamethasone in Plasma by High-Performance Liquid Chromatography with Tandem Mass Spectrometry and Its Application to a Pharmacokinetic Comparison between Normoxic and Hypoxic Rats

**DOI:** 10.3390/molecules20046901

**Published:** 2015-04-16

**Authors:** Wenwen Gong, Shuhong Liu, Pingxiang Xu, Ming Fan, Ming Xue

**Affiliations:** 1 Department of Neurobiology, School of Basic Medical Sciences, Capital Medical University, Beijing 100069, China; E-Mail: gongww12345@126.com; 2 Department of Pharmacology, School of Basic Medical Sciences, Capital Medical University, Beijing 100069, China; E-Mail: syxpx88@163.com; 3 Department of Cognitive Sciences, Beijing Institute of Basic Medical Sciences, Beijing 100850, China; E-Mail: riever62002@126.com; 4 Beijing Laboratory for Biomedical Detection Technology and Instrument, Department of Pharmacology, School of Basic Medical Sciences, Capital Medical University, Beijing 100069, China

**Keywords:** LC-MS/MS, diazepam, dexamethasone, pharmacokinetics, hypoxia

## Abstract

In order to investigate the pharmacokinetics of a combination of diazepam and dexamethasone under hypoxic conditions, a novel, sensitive and specific liquid chromatography with tandem mass spectrometry (LC-MS/MS) method for the simultaneous determination of diazepam and dexamethasone in rat plasma was developed and validated. The chromatographic separation of analytes was successfully achieved on an XTerra^®^ MS C_18_ column using a gradient elution of methanol and water containing 0.1% formic acid at a flow rate of 0.5 mL/min. This method demonstrated good linearity and no endogenous material interferences. The linear ranges were 1.0–100 ng/mL for diazepam and 2.0–200 ng/mL for dexamethasone. The intra- and inter-day precision for the two compounds in plasma were lower than 10.0%, and the accuracy was between −7.9% and 11.5%. Our method was then successfully applied in a pharmacokinetic comparison between normoxic and hypoxic rats. The results indicated that there were significant differences in the main pharmacokinetics parameters of diazepam and dexamethasone between normoxic and hypoxic rats. The results provide the important and valuable information for discovering and developing novel anti-hypoxia drug combinations, as well as a better understanding of the safety and efficacy of these drugs.

## 1. Introduction

Acute mountain sickness (AMS) is a dangerous hypoxic illness that can affect humans who rapidly reach a high altitude above 3000 m [1]. Hypoxia usually causes peripheral vasoconstriction and increased venous return and blood flow, resulting in pulmonary vascular contraction, eventually leading to hypoxic cerebral and pulmonary hypertension. In severe cases high altitude cerebral edema as well as pulmonary edema happen to the patients with AMS [2]. Hypoxia induced at high altitude also causes a subnormal oxygen concentration in cells which affects the drug metabolic and pharmacokinetic capacity [3,4,5]. Several studies have indicated that hypoxia alters the pharmacokinetic characteristics of some drugs. Patients with chronic obstructive lung disease (COLD), pulmonary edema, pulmonary heart disease or congestive heart failure show significantly reduced theophylline clearance [6]. Compared with the data without hypoxia, the half-life of antipyrine was increased by 120% in patients with chronic hypoxemia, while the half-life of tolbutamide was decreased by 33% in patients with chronic asthma exposed to hypoxic conditions and tolbutamide clearance was increased by 180% in hypoxic subjects compared with controls [3]. 

**Figure 1:**
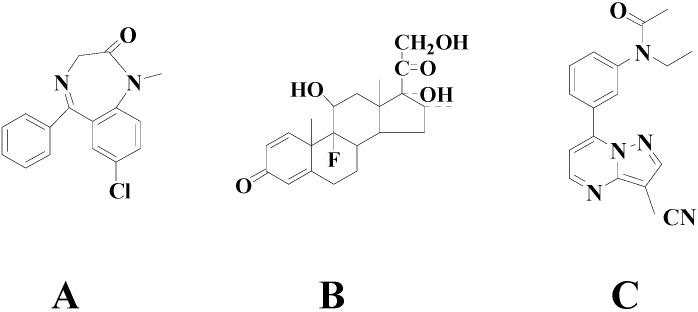
Chemical structures of the analytes: diazepam (**A**), dexamethasone (**B**) and the internal standard (IS) zaleplon (**C**).

Diazepam and dexamethasone (Figure 1) are currently used clinically on the plateau at the same time. They demonstrate a variety of pharmacological activities, including effectively relieving cerebral edema and pulmonary congestion, stabilizing emotions, reducing the body’s oxygen consumption, improving ventilation, effective dieresis, and preventing water sodium retention [7,8]. Previous studies indicated that diazepam and dexamethasone had significant anti-hypoxic effects at high altitude by improving lung tissue oxidative imbalances [9,10]. Diazepam is widely used as a muscle relaxant, sedative, anxiolytic or anticonvulsant. Dexamethasone can enhance acclimatization to hypoxia by improving oxygenation and indirectly lowering pulmonary arterial pressure [11,12,13]. The analytical methods for determination of diazepam [14,15] or dexamethasone [16,17,18] alone in biological fluids have been reported, while to date, there is still no report about an *in vivo* simultaneous quantification of diazepam and dexamethasone. In this paper, we first developed a novel, sensitive and specific method for the simultaneous quantification of diazepam and dexamethasone in rat plasma, and then successfully applied it to study the pharmacokinetics of diazepam and dexamethasone in normoxic and hypoxic rats. The results provide an important and valuable information for discovering and developing novel anti-hypoxia drug combinations, as well as a better understanding of the safety and efficacy of these two drugs.

## 2. Results and Discussion

### 2.1. Method Development

Protein precipitation was tested for sample preparation in our test. Methanol as precipitant could provide a satisfactory recovery, and this method proved to be simple and suitable for simultaneous determination of these two components in rat plasma. Prolonged retention time is beneficial in analysis of these plasma samples, to avoid co-elution with early eluting endogenous compounds that cause ion suppression. The XTerra^®^ MS C_18_ column (Waters, Milford, MA, USA) plus C_18_ guard column were evaluated and proved to be suitable for simultaneous separation of these compounds. The concentration of aqueous 0.1% (*v*/*v*) formic acid solution was proved to give a better response than other aqueous phases (e.g. for 0.2% formic acid). Gradient elution changed linearly from 75:25 (*v*/*v*) 0.1% formic acid aqueous solution-methanol to 30:70 at a flow rate of 0.5 mL/min. Compared with some compounds (such as verapamil, reserpine, *etc*.) we tested, the IS zaleplon had a more suitable retention time in the chromatographic separation, the IS had the similar chemical properties as well as efficiency for the ESI ionization of the analytes.

Electrospray ionization (ESI) was adopted to quantify the analytes in rat plasma due to its lower levels of background noise than APCI. Positive ion mode was found to provide better sensitivity for the active compounds. The capillary temperature, vaporizer temperature and flow rate were optimized to obtain protonated molecules of the analytes. The fragmentation energy was optimized to achieve maximum response of the compound fragment ion peaks. SRM was used for the simultaneous quantification of the two analytes and the quantitative ions were chosen because all of the product ions were the most abundant ion. 

### 2.2. Method Validation

#### 2.2.1. Selectivity

**Figure 2:**
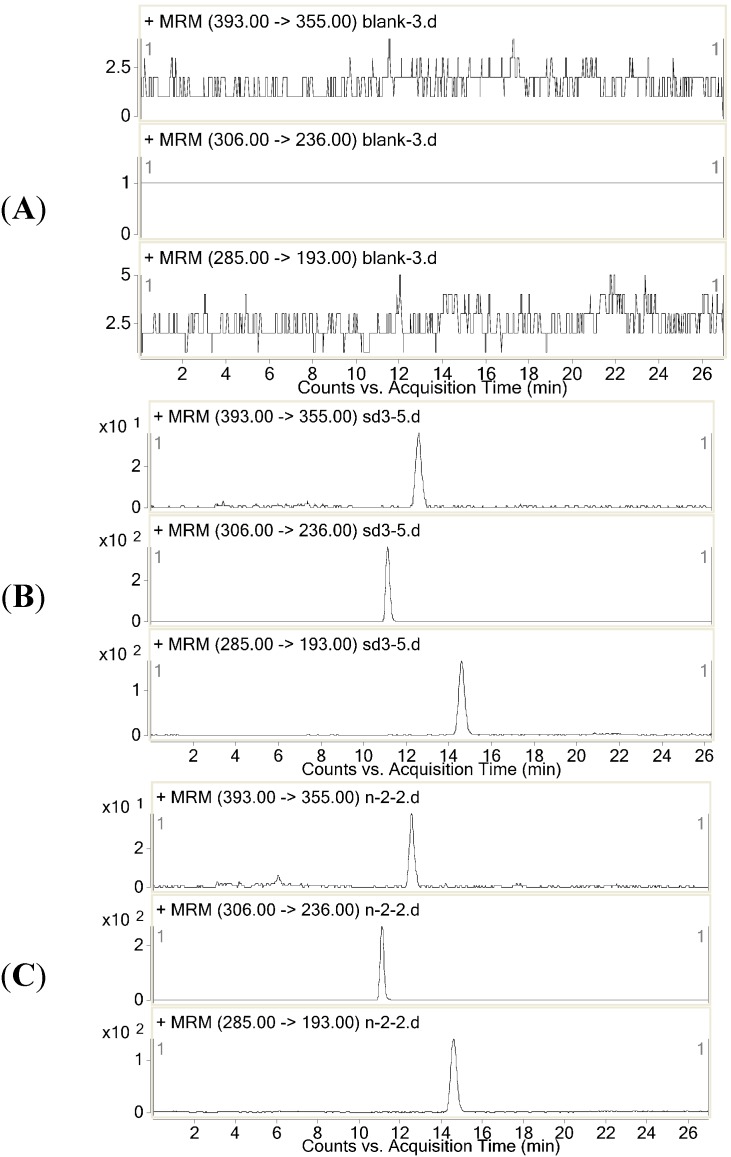
Representative SRM chromatography of (**A**) a plasma sample; (**B**) a blank plasma spiked with diazepam, dexamethasone and the IS; (**C**) a rat plasma sample collected 0.5 h after drug administration.

Selectivity was investigated by analyzing blank samples from five different batches of rat plasma. The representative SRM chromatograms of the diazepam and dexamethasone and the selectivity of these two drugs are shown in Figure 2. The results showed that there were no endogenous substance peaks from the rat plasma samples and drug metabolite peaks interfering with the analytes and the IS at the retention times. The LC-MS/MS method described was thus selective and specific.

#### 2.2.2. Linearity and Lower Limit of Quantification

Quantification was based on the IS method of plotting the peak areas ratios of the analyte/IS *versus* the nominal plasma concentration of the test compound with 1/*x*^2^ as weighting factors, which was fitted by least square linear regression. The regression equations of these analytes were the following: diazepam: *y* = 5.114*x* + 0.0104 (*r*^2^ = 0.992) and dexamethasone: *y* = 0.4888*x* + 0.0059 (*r*^2^ = 0.994). The calibration curves were linear from 1.0 to 100 ng/mL for diazepam and from 2.0 to 200 ng/mL for dexamethasone.

Sensitivity was evaluated by determining the lower limit of quantification (LLOQ), which are defined as the lowest concentration that can be reliably and reproducibly measured at least five replicates. The LLOQ had to have precision of ≤20% and a signal/noise ratio ≥10. The LLOQ data from the chromatograms of the blank samples that spiked with the analytes were 1.0 ng/mL for diazepam and 2.0 ng/mL for dexamethasone, respectively. 

#### 2.2.3. Precision and Accuracy

The precision and accuracy of the method were assessed in rat plasma by performing replicate analyses of QC samples. The procedure was repeated on the same day and between three different days on the same spiked standard series. The data are shown in Table 1, indicating that the precision and accuracy of the method are acceptable.

**Table 1:** Precision and accuracy of diazepam and dexamethasone in rat plasma.

Compound	Spiked	Intra-day ( *n* = 6)			Inter-day ( *n* = 18)		
		Measured (mean ± SD) (ng/mL)	Precision (RSD) %	Accuracy (%)	Measured (mean ± SD) (ng/mL)	Precision (RSD) %	Accuracy (%)
Diazepam	2.0	2.1 ± 0.20	9.5	5.9	2.0 ± 0.19	9.7	0.9
	10	11.2 ± 0.33	2.9	11.5	11.0 ± 0.30	2.7	10.1
	50	55.6 ± 1.1	2.0	11.2	53.2 ± 4.0	7.6	6.5
Dexamethasone	4.0	4.1 ± 0.41	10.0	2.4	4.0 ± 0.39	10.0	−0.85
	20	19.3 ± 1.9	10.0	−3.4	19.7 ± 1.8	9.0	−1.7
	100	92.7 ± 3.0	3.3	−7.3	92.1 ± 5.3	5.8	−7.9

#### 2.2.4. Recovery and Matrix Effect

The recoveries were determined by analyzing six replicates of QC samples. The results are summarized in Table 2. The mean recovery of IS in rat plasma was 110% ± 5.2%. The data indicated that the recoveries of the analytes and IS from the rat plasma were concentration-independent in the concentration range evaluated and the recoveries were acceptable for the pharmacokinetic analysis. 

**Table 2:** The recovery and matrix effect of diazepam and dexamethasone in rat plasma.

Compound	Spiked Concentration (ng/mL)	Recovery (mean ± SD) % ( *n* = 6)	RSD (%)	Matrix Effect (mean ± SD) % ( *n* = 3)	**RSD (%)**
Diazepam	2.0	98.4 ± 5.2	5.2	92 ± 9.6	10.4
	10	102 ± 1.2	1.2	112 ± 1.3	1.2
	50	94.5 ± 1.8	2.0	109 ± 1.3	1.2
Dexamethasone	4.0	103 ± 9.0	8.7	113 ± 4.8	12.9
	20	107 ± 10	9.4	109 ± 3.6	9.9
	100	99.2 ± 3.6	3.6	99 ± 2.2	6.7

We investigated the matrix effect in rats from three different sources, all the results were in the range of 92%–113%, and the IS showed 103%, indicating that no latent co-eluting endogenous substance interfered with the ionization of analytes and IS. The matrix effect of two analytes in rat plasma was shown in Table 2.

#### 2.2.5. Stability

The stability study showed that the variation in the concentration was within ±15% of the nominal concentration (Table 3), indicating no significant degradation of any analyte occurred in rat plasma after storage for 30 days at −80 °C, post treatment storage for 24 h at room temperature, or three freeze-thaw cycles. 

**Table 3:** Stability of diazepam and dexamethasone in the QC sample.

Compound	Concentration Spiked (ng/mL)	Storage for 30 days at −80 °C		Post Treatment Storage for 24 h at RT *		Three Freeze-Thaw Cycles	
		Concentration Measured (ng/mL) ( *n* = 3)	RSD (%)	Concentration Measured (ng/mL) ( *n* = 3)	RSD (%)	Concentration Measured (ng/mL) ( *n* = 3)	RSD (%)
Diazepam	2.0	2.0 ± 0.24	11.9	2.02 ± 0.07	3.4	1.86 ± 0.20	10.6
	10	11.1 ± 0.15	1.4	11.1 ± 0.26	2.4	11.0 ± 0.30	2.8
	50	56.1 ± 0.97	1.7	55.9 ± 0.55	1.0	56.3 ± 0.92	1.6
Dexamethasone	4.0	4.2 ± 0.25	5.9	4.13 ± 0.32	7.8	3.7 ± 0.30	8.1
	20	21.5 ± 1.6	7.5	19.8 ± 2.4	12.3	20.2 ± 1.3	6.3
	100	91.2 ± 6.8	7.5	87.6 ± 2.6	2.9	91.0 ± 5.6	6.2

* RT, Room temperature.

### 2.3. Pharmacokinetics Comparison

**Figure 3:**
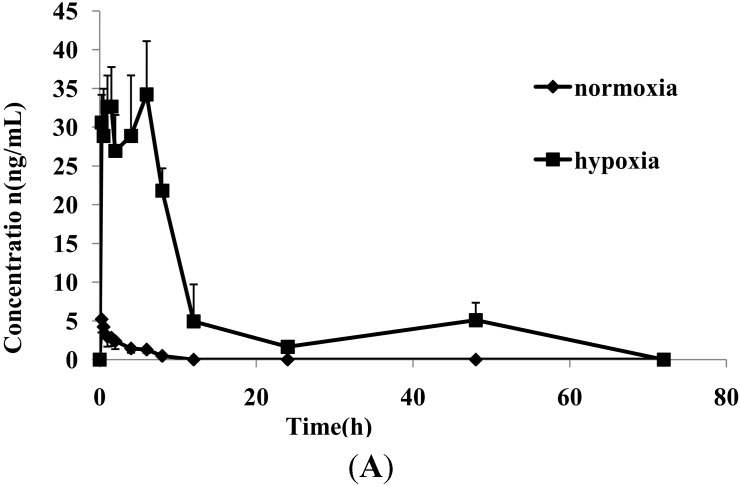

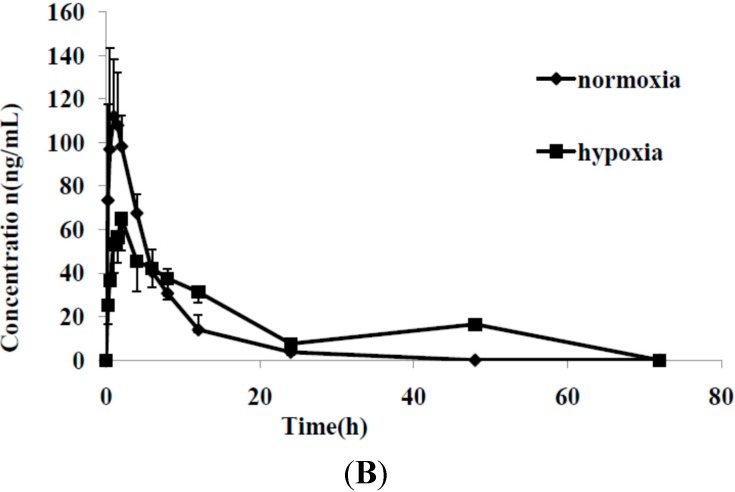
Mean plasma concentration-time curves of diazepam (**A**) and dexamethasone (**B**) in rat with and without hypoxia following intragastric administration to rats (*n* = 6).

The LC-MS/MS method showed satisfactory results for the simultaneous determination of diazepam and dexamethasone in rat plasma. This method was then successfully used for comparing the pharmacokinetic characteristics of these two drugs between normoxic and hypoxic rats. Figure 3 shows the mean plasma concentration-time profiles of diazepam and dexamethasone under normoxic and hypoxic conditions. The changes in the main pharmacokinetic parameters of diazepam influenced by hypoxia are shown in Table 4. From a comparison of the main pharmacokinetic parameters of diazepam between normoxic and hypoxic rats, the peak plasma concentration (C_max_), time to reach C_max_ (T_max_), area under the plasma drug concentration-time curve (AUC) and mean residence time (MRT) of diazepam in hypoxic rats were markedly increased, and there were statistically significant differences in these parameters (*p* < 0.01 or *p* < 0.05).

**Table 4:** Pharmacokinetic parameters of diazepam and dexamethasone to rats with and without hypoxia (*n* = 6).

Parameter	Diazepam		Dexamethasone	
	Normoxia	Hypoxia	Normoxia	Hypoxia
C_max_ (ng/mL)	5.3 ± 1.6	39.2 ± 4.3 **	121 ± 31.1	65.7 ± 13.9 **
T_max_ (h)	0.30 ± 0.11	3.1 ± 2.6 *	1.3 ± 0.57	2.17 ± 0.931
*k*	0.38 ± 0.077	0.27 ± 0.18	0.21 ± 0.063	0.072 ± 0.027 **
MRT (h)	2.3 ± 0.51	6.5 ± 2.2 **	4.3 ± 1.2	9.14 ± 3.44 *
t_1/2_ (h)	4.2 ± 3.7	3.3 ± 1.4	3.5 ± 0.94	11.5 ± 6.6 *
AUC_0→72_ (ng/mL∙h)	13.0 ± 4.3	326 ± 39.6 **	656 ± 141	750 ± 232

Data are given as the mean ± SD; * *p* < 0.05 and ** *p* < 0.01 showed significantly different between the normoxic and hypoxic groups.

The values of AUC and C_max_ of diazepam in hypoxia were increased by 25 and 7.4 times, respectively, indicating that the *in vivo* content and level of diazepam increased markedly. Figure 3 shows that diazepam had double peaks, suggesting that reabsorption occurred, which may be associated with the hypoxic exposure. The results show that the absorption of diazepam in hypoxic rats was markedly faster, and the elimination was markedly slower than in normoxic rats, strongly indicating that hypoxia significantly altered the pharmacokinetics characteristics of diazepam in rats.

The pharmacokinetic parameters of dexamethasone are shown in Table 4. Compared with the control group, hypoxia significantly increased the values of some pharmacokinetic parameters of dexamethasone including the t_1/2_ (*p* < 0.05) and MRT (*p* < 0.05), and markedly decreased the *k* (*p* < 0.01) and C_max_ (*p* < 0.01). The results showed that the elimination of dexamethasone in hypoxic rats was markedly slower than in normoxic rats, indicating that hypoxia significantly altered the main pharmacokinetics characteristics of dexamethasone in rats. These changes in the pharmacokinetic parameters of these two drugs were in favor of treatment of some diseases related hypoxia. The results provide the important and valuable information for better understanding of the safety and efficacy of these two drugs in clinical practice [19].

## 3. Experimental Section

### 3.1. Chemicals and Reagents

Diazepam, dexamethasone and the internal standard (IS) zaleplon were purchased from National Institute for the Control of Pharmaceuticals and Biological Products (Beijing, China). HPLC-grade methanol was obtained from Thermo Fisher Scientific (Waltham, Australia). HPLC grade formic acid was purchased from Dikma Reagent Company (Beijing, China). Water used in the experiment was double distilled.

### 3.2. Animals

Male Sprague-Dawley rats (250 ± 20 g) were purchased from the Animal Center of Capital Medical University (ACCMU, Beijing, China). Animals were housed in individual cages with free access to food and water in a room with an automatically controlled illumination (a 12 h light-dark cycle), temperature and relative humidity. Animal studies were carried out in accordance with the Guide for the Care and Use of Laboratory Animals as adopted and promulgated by the National Health Ministry of China. Protocols of animal experiments had been approved by Animal Center of Capital Medical University.

Six rats were exposed to a fractional concentration of inspired O_2_ (FiO_2_) of 9.0% after intragastric administration of drugs. Five rats were exposed to the normal air condition as the normoxic groups.

The blood samples were collected in tubes containing sodium heparin at 0, 0.25, 0.5, 1, 1.5, 2, 4, 6, 8, 12, 24, 48 and 72 h after dosing (dexamethasone: 0.34 mg/kg, diazepam: 1.1 mg/kg), the rat dose was converted from the drugs through the human doses in clinical practice. The plasma samples were taken after centrifugation at 3000 rpm for 10 min and stored at −80 °C until analysis.

### 3.3. LC-MS/MS Conditions

The LC-MS/MS system consisted of a HPLC system (Agilent Technologies, Palo Alto, CA, USA) including a HP G1312A binary pump, a G1379A vacuum degasser and G1313A autosampler and triple quadrupole mass spectrometer equipped with electrospray source (Series 6410, Agilent Technologies).

The analytes were separated on a Xterra^®^ MS C18 column (4.6 mm × 150 mm, 5 μm), protected with ZORBAX Eclipse plus C18 guard column (2.1 mm × 12.5 mm, 5 μm). The mobile phase solutions were composed of water containing 0.1% (*v*/*v*) formic acid (A) and methanol (B). Initial gradient conditions were 75:25 (A:B). From the 0 to 5 min the B was increased to 35%. From 5 to 7 min the B was increased to 70%. The total run time was 16 min. The mobile phase was returned to the initial conditions and reequilibration for a period of time. The flow rate was 0.5 mL/min. The sample injection volume was 10 μL.

The MS ionization source conditions were as follows: capillary voltage of 4.0 kV, drying gas temperature of 300 °C, drying gas flow: 10 L/min, nebulizer pressure: 45 psi and corona current 10 nA. The sheath gas flow was 7 L/min and sheath gas temperature was 250 °C. The positive ion modes were performed with SRM for the quantitative analysis by ESI. The SRM quantitative ions were then selected from the MS/MS data. The optimized precursor-to-product ion transitions were monitored for diazepam [M+H]^+^*m*/*z* 285→193 with fragmentor 140 V and collision energy (CE) 32 V, dexamethasone [M+H]^+^*m*/*z* 393→355 with fragmentor 110 V and CE 5 V and IS [M+H]^+^*m*/*z* 306→236 with fragmentor 155 V and CE 25 V, respectively.

### 3.4. Preparation of Calibration Standards and Quality Control Samples

Stock solutions of diazepam (1 μg/mL), dexamethasone (2 μg/mL) and the IS (2 μg/mL) were prepared in methanol, respectively. Different amounts of each stock solution were mixed and diluted with methanol to prepare the series of working solutions for the analytes. In addition, the stock solution of the IS was also diluted to the concentration of 200 ng/mL with methanol. All the solutions were kept at 4 °C prior to use. Calibration standards were prepared by spiking the appropriate amount of the mixture standard working solution into the blank rat plasma to give the nominal concentration range of 1.0–100 ng/mL for diazepam and 2.0–200 ng/mL for dexamethasone. Three levels of QC samples (2.0, 10 and 50 ng/mL for diazepam and 4.0, 20 and 100 ng/mL for dexamethasone) in plasma were prepared in the same way as the calibration standards.

### 3.5. Samples Preparation

For plasma sample preparation, a plasma sample (50 μL) was placed into a 1.5 mL Eppendorf tube. After the addition of 50 μL of 200 ng/mL solution of IS, the tube was briefly mixed and 100 μL methanol were added into the tube. After vortex mixing for 30 s, the samples were centrifuged at 13,000 rpm for 10 min. The supernatant was then transferred into autosampler vial, and 10 μL aliquot was injected in to the LC-MS/MS system for analysis.

### 3.6. Method Validation

#### 3.6.1. Selectivity and Specificity

To evaluate the selectivity, the independent samples of rat blank plasma were analyzed by comparing with the plasma-spiked analytes for excluding endogenous material interference. Specificity refers to the ability of analytical method to differentiate and quantify the analytes in the presence of other components. Specificity was examined by applying the pretreatment procedure to drug-free rat plasma samples (*n* = 5) as previously described.

#### 3.6.2. Linearity and Lower Limit of Quantification

The concentration of each analyte was determined by using the equations of linear regression obtained from the calibration curves. To assess linearity, deviation of the mean calculated concentration over three runs should be within ±15% of nominal concentration with the RSD ≤ 15%, the mean value determined at LLOQ should not deviate by more than ±20% of the actual value, and the precision determined at LLOQ should not exceed ±20% of the RSD.

#### 3.6.3. Precision and Accuracy

The precision and accuracy of the assay were assessed by analyzing QC samples in six replicates on the same day and three different days for intra- and inter-day precision and accuracy, respectively. Acceptable limits for intra- and inter-day precision and accuracy were set at ±15%.

#### 3.6.4. Recovery and Matrix Effects

Recoveries of diazepam and dexamethasone were determined by comparing the peak areas of the extracted QC samples with the peak areas of post-extracted blank plasma spiked at the corresponding concentrations. The recovery of the IS was determined in the same way at the concentration of 200 ng/mL.

The matrix effect was evaluated by comparing the peak areas of the post-extracted blank plasma spiked with the working solutions with those of corresponding standard solutions. An analogous procedure was applied to the IS.

#### 3.6.5. Stability

The stability of the sample was assessed by measuring the analysis data of QC samples under ambient, frozen and freeze-thaw storage conditions with fresh prepared QC samples. The long-term stability was determined after 30 days at −80 °C and freeze-thaw stability was determined after three freeze (−80 °C) and thaw (room temperature) cycles. To assess autosampler stability, the extracts were placed in the autosampler at room temperature for 24 h prior to analysis. An acceptable stability was defined as ≤15% loss of the initial drug concentration.

### 3.7. Pharmacokinetics Comparison

The pharmacokinetic analysis was performed using the noncompartmental model analysis. The peak plasma concentrations (C_max_) of diazepam and dexamethasone, and the time to reach peak plasma concentration (T_max_) were obtained by inspection of the individual concentration time data. The terminal elimination rate constant (k) was estimated by linear regression of the terminal portion of the concentration-time curve, and the elimination half-life (t_1/2_) was calculated as 0.693/k. The mean residue time (MRT) was the time to eliminate 63.2% drug in the body. The area under the plasma concentration-time curves (AUC) of diazepam and dexamethasone were calculated by the integral calculation.

### 3.8. Data Analysis

All measurement values are reported as the mean ± standard deviation (SD). Comparisons between the groups of rats were performed using independent-samples *t*-test. The SPSS 18.0 (SPSS Inc., Chicago, IL, USA) was used for data analysis. A *p* value < 0.05 was considered statistically significant.

## 4. Conclusions

A novel LC-MS/MS method for the simultaneous determination of diazepam and dexamethasone in rat plasma was developed for the first time, and successfully applied to characterize and compare the pharmacokinetics of these two drugs in normoxic and hypoxic rats. Our results indicated that hypoxia significantly altered the *in vivo* pharmacokinetic characteristics of diazepam and dexamethasone after oral administration of the drugs in rats. These changes in the pharmacokinetic parameters were in favor of treatment of some effects related to hypoxia. These results provide important and valuable information for discovering and developing a novel anti-hypoxia drug combination, as well as a better understanding of the safety and efficacy of diazepam and dexamethasone in clinical practice.
